# HIV Infection and Host Genetic Mutation among Injecting Drug-users of Northeastern States of India

**DOI:** 10.3329/jhpn.v28i2.4882

**Published:** 2010-04

**Authors:** Kamalesh Sarkar, Santa Sabuj Das, Reshmi Pal, Baishali Bal, P. Madhusudan, Sekhar Chakraborti

**Affiliations:** National Institute of Cholera & Enteric Diseases, P-33 CIT Road, Scheme XM, Kolkata 700 010, India

**Keywords:** Community-based studies, Cross-sectional studies, Genetic mutation, HIV infections, Sexually transmitted diseases, Substrance-use, India

## Abstract

A community-based cross-sectional study was conducted among injecting drug-users (IDUs) of the northeastern states of India to understand the host genetic factors that confer resistance to HIV infection. The study aimed at assessing the existence and magnitude of genetic mutations of chemokine receptors, such as CCR2-64I, CCR-5 D-32, and SDF-1-3‘A, that are known to confer resistance to HIV infection and progression of disease in some set-ups. In total, 711 IDUs from Manipur, Mizoram, Nagaland, and Meghalaya were sampled for the study. The selected participants were interviewed to study their sociodemography, risk behaviours, and risk perceptions after obtaining their verbal informed consent. The interview was followed by collection of about 5 mL of blood samples by an unlinked anonymous method for studying genetic mutation and HIV infection. All the blood samples were transported to and processed at the clinical medicine laboratory of the National Institute of Cholera & Enteric Diseases, Kolkata, India. The genetic mutations were detected by polymerase chain reaction (PCR) and the restriction fragment length polymorphism (RFLP) assay techniques. The study revealed that 328 (46.1%) IDUs were aged 20–29 years, 305 (42.9%) were aged 30–39 years, and only two (0.3%) were aged above 49 years. The rate of HIV seropositivity varied widely among the IDUs living in different northeastern states that ranged from 4.5% to 61%. There was not a single IDU with CCR5 homozygous mutation. Mutated genes of CCR2-64I and SDF-1-3'A were detected in the frequencies of 49% and 23% respectively in them. The rate of HIV seropositivity in IDUs having CCR2 mutant gene was 27% (n=94) and without mutation was 27% (n=98). Similarly, HIV seropositivity in IDUs with and without SDF1 mutation was 28% (n=46) and 27% (n=146) respectively. Both the differences were not statistically significant. A CCR5 homozygous mutation is known to be the most prominent marker that confers resistance against HIV infection. The absence of CCR5 mutant gene in this population suggests that they do not have any additional protection against HIV infection. Analysis also revealed that, although mutation of CCR2 and SDF1 was present in this population, it did not confer any additional resistance against HIV. This indicates that the IDUs of northeastern India are not additionally protected against HIV infection through genetic mutation and are, therefore, vulnerable to acquire HIV infection due to high-risk behaviour and other related factors.

## INTRODUCTION

HIV continues to be an important public-health problem in India since its first detection in 1986. The national-level prevalence of HIV among adults in India is around 0.3%, which gives rise to an estimated 2–3.6 million HIV-infected people in the country (1). The genetic differences between individuals appear to be an essential factor towards protection against HIV infection despite indulgence in high-risk behaviour (2). The risk factors associated with the development of clinical disease and the life-span of HIV infection in individuals are less understood.

Individuals with variants of the genes encoding the chemokine receptors—CCR2 and CCR5—and the ligand SDF1 have been shown to be resistant to HIV-1 infection and progression of disease (3–6). Of the three genetic markers, presence of homozygous CCR5 D-32 allele appears to be the most important factor that confers resistance against HIV-1 infection, and heterozygous mutation prevents the progression of disease (7). The natural resistance to HIV-1 infection has been described by two mechanisms: one is termed ‘exposed-uninfected’ and the other one ‘long-term non-progressors’. In the former type, individuals are exposed repeatedly over a long period without any manifestations of HIV infection. Such individuals include commercial sex workers, people having unprotected sex with infected partners, infants of HIV-positive mothers, IDUs, haemophiliacs, etc. The latter types of individuals carry HIV virus which either does not progress or progresses at a very slow rate. Homosexuals, IDUs, infants, or children usually fall in this group as described by Marmor *et al.* (8). Such facts have provided several options for research and development of more and more safe and effective drugs and vaccines to combat HIV infection at a very early stage of its pathogenesis.

Of all the northeastern states of India, Manipur in particular is contributing to the rapid spread of HIV-seropositive cases among IDUs (9). Although sharing of injecting equipment and paraphernalia is the main mode of transmission among IDUs here, sexual transmission is also currently increasing there (10). Transmission of HIV in local IDUs has always been viewed as an interaction between behavioural or cultural practices and presence of circulating HIV in the community. Influence of host factors, particularly genetic susceptibility, as mentioned above, has never been thought of and investigated in the said population. Hence, a study was conducted among IDUs of northeastern India to understand the existence and magnitude of genetic variants encoding CCR5, CCR2, and SDF1 and their relationships with the prevalence of HIV infection among them.

## MATERIALS AND METHODS

This community-based cross-sectional study was conducted among 711 IDUs from four northeastern states: Manipur, Nagaland, Mizoram, and Meghalaya. Initially, the purpose of the study was explained to all the participants who were invited to participate voluntarily after obtaining their verbal consent. Ethical clearance was obtained from the ethical committee of the National Institute of Cholera & Enteric Diseases (NICED) before initiation of the study. Two experienced social workers interviewed the study participants using a field-tested questionnaire to study their sociodemography, risk behaviour, and risk perceptions about HIV infection. The interview was followed by collection of about 5 mL blood sample by an unlinked anonymous method for testing HIV and genetic mutation. All the samples were then transported to and processed at the clinical medicine laboratory of the NICED, a national AIDS reference laboratory. The genetic mutations were detected by polymerase chain reaction (PCR) and the restriction fragment length polymorphism (RFLP) assay techniques as described below.

Primers for the amplification of CCR5 (FP: 5’ TTA AAA GCC AGG ACG GTC AC 3’ and RP: 5’ TGT AGG GAG CCC AGA AGA GA 3’), CCR2 (FP: 5’ TTG TGG GCA ACA TGA TGG 3’ and RP: 5’ CTG TGA ATA ATT TGC AGA TTG C 3’), and SDF1 (FP: 5’ AAG GCT TCT CTC TGT GGG ATG 3’ and RP: 5’ GAC AGT CGT GGA CAC ACA TGA T 3’) were custom-synthesized from Integrated DNA Technologies (IDT), Inc., USA. DNA was extracted from peripheral blood samples following the standard procedure. Peripheral blood mononuclear cells (PBMCs) were isolated from 100 to 200 μL of blood by pelleting down the cells at 1,000 rpm for five minutes. Red blood cells (RBCs) were lysed by re-suspending the cell pellet in 500 μL of ACS-buffered saline, followed by incubation at room temperature for 3–4 minutes. Cell suspension was centrifuged at 6,000 rpm for five minutes after the addition of 500 μL of RPMI medium. The pellet containing PBMCs was washed with 500 μL of PBS and lysed by the addition of 500 μL of DNAZOL (invitrogen-USA). 0.25 mL of 100% ethanol was then added to the lysate, and genomic DNA was spooled out with a pipette-tip and transferred to a fresh tube. DNA was washed twice with 0.8–1.0 mL of 75% ethanol and dissolved in 100 μL 8 mM NaOH. PCR amplification of the genes under study was done in a 50-μL reaction containing 5 μL of 10×PCR buffer, 1 μL of dNTPs (2.5 mM stock), 0.4 μL of each primer (10 μM stock), ng of genomic DNA, and 2.5 U of Taq DNA polymerase. For the amplification of CCR5, the reaction mixture was subjected to denaturation at 94°C for five minutes, followed by 35 cycles of denaturation at 94°C for 45 seconds, annealing at 62°C for 30 seconds, and extension at 72°C for one minute. For CCR2, initial denaturation was followed by 35 cycles of denaturation at 94°C for one minute, annealing at 56°C for 45 seconds, and extension at 72°C for one minute. SDF-1 was also amplified for 35 cycles (denaturation at 94°C for one minute, annealing at 50°C for one minute, and extension at 72°C for 1.30 minutes). A final primer extension at 72°C for five minutes was allowed in each case before the PCR was stopped. To analyze RFLP for SDF1-3'A, PCR products were digested with MspI restriction enzyme at 37°C for 16 hours. RFLP for CCR2-64I was analyzed by digesting the PCR product with BsaB1 for four hours at 65°C. The digested products were resolved in 3% agarose gel. All the data, including laboratory test results, were edited, entered, and analyzed using the Epi Info software (version 6.04).

## RESULTS

**Fig. 1.**
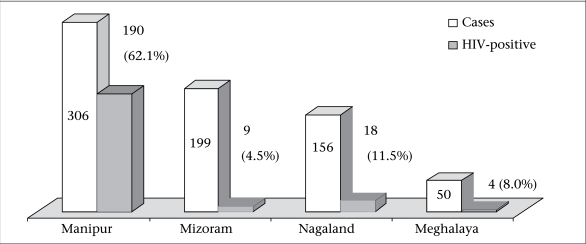
State-wise participation of IDUs with HIV seropositivity

In total, 711 IDUs from four different northeastern states participated in the study. Figure 1 shows that Manipur had the highest number (n=306, 43%) of the study participants, followed by Mizoram 28% (n=199), Nagaland 21.9% (n=156), and Meghalaya 7% (n=50). The HIV seropositivity was 62.1% (n=190), 4.5% (n=9), 11.5% (n=18), and 8% (n=4) in IDUs of Manipur, Mizoram, Nagaland, and Meghalaya respectively.

**Fig. 2.**
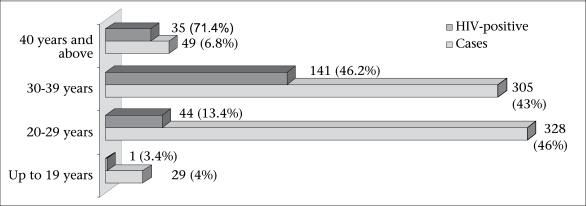
Age-wise distribution of IDUs (n=711)

Three hundred twenty-eight (46%) participants were aged 20–29 years, 305 (43%) were aged 30–39 years, and 29 (4%) were aged 19 years or less. HIV seropositivity showed an increasing trend with the increase in age (Fig. 2).

In terms of gender distribution, 94% (n=668) were male participants while 6% (n=43) were females. However, the rate of HIV seropositivity in female IDUs was more when compared with their male counterparts (females=65.1%; n=43 and males=28.9%; n=668). About half (51.2%; n=364) of them were unmarried, and 34.7% (n=247) were married. About 6.5% (n=46) were married but living separately without legal separation. The remaining 3.7% (n=26) and 3.9% (n=28) were widows and divorcees respectively. Half (50.5%; n=359) of the study population was literate up to the level of secondary education, followed by primary education (17.3%; n=123). About 36% (n=255) of the study subjects were unemployed. Other occupation-groups included service-holders, labourers, students, and businessmen in varying frequencies. Spasmoproxyvon was the most frequently-used substance (59%; n=419), followed by heroin (54%; n=384) and brown sugar (15%; n=109).

A sizable number of the IDUs shared their injecting equipment and paraphernalia. Transmission of HIV infection is known to be associated with sharing unsafe injection. Table 1 shows that 53.3% (n=379) of the participants shared their injecting equipment either always or frequently, and the rate of HIV seropositivity in them was 46.7% (n=177). On the other hand, 46.6% (n=332) shared either occasionally or never, with an HIV-seropositivity rate of 13.2% (n=44). This difference was significant as indicated by odds ratio (OR) of 5.7, with a 95% confidence (CI) level of 3.8–8.5.

**Table 1.** Risk behaviour relating to sharing of injecting equipment/paraphernalia and HIV infection (n=711)

Particulars	Cases		HIV-positive		Odds ratio (95% CI)	p value
	No.	%	No.	%		
Frequency of sharing injecting equipment						
Always/frequently	379	53.3	177	46.7	5.7 (3.8–8.5)	<0.05*
Occasionally/never	332	46.6	44	13.2		
Sharing of drugs from common ampoules						
Yes	237	33.3	150	63.3	9.79 (6.6–14.3)	<0.05*
No	474	66.7	71	15.0		
Sharing of water for cleaning syringes before injection						
Yes	443	62.3	189	42.7	5.4 (3.5–8.4)	<0.05*
No	268	37.7	32	11.9		
Sharing of used cotton to stop bleeding after injection						
Yes	152	21.4	62	40.8	1.73 (1.1–2.5)	<0.05*
No	559	78.6	159	28.4		

*Significant

In total, 237 (33.3%) of the IDUs shared drugs from common ampoules, with an HIV-seropositive rate of 63.3% (n=150), and 66.7% (n=474) did not have a similar history of sharing. The HIV-seropositive rate was 15% (n=71) in them. This difference was significant as indicated by OR of 9.7 and 95% CI level of 6.6–14.3. 62.3% (n=443) of the participants shared water for cleaning their syringes before injecting, and the rate of HIV seropositivity was 42.7% (n=189) in them. On the contrary, 37.7% (n-268) did not share, and the rate of HIV sero positivity was 11.9% (n=32). This difference was significant indicated by OR of 5.4 and 95% CI level of 3.5–8.4. 21.4% (n=152) shared cotton to stop bleeding following injection with an HIV-seropositivity rate of 40.8% (n=62), 78.6% (n=559) did not share, and the rate of HIV seropositivity was 28.4% (n=159) in them. The difference was significant with OR of 1.7 and 95% CI level of 1.1–2.5 (Table 1).

**Fig. 3.**
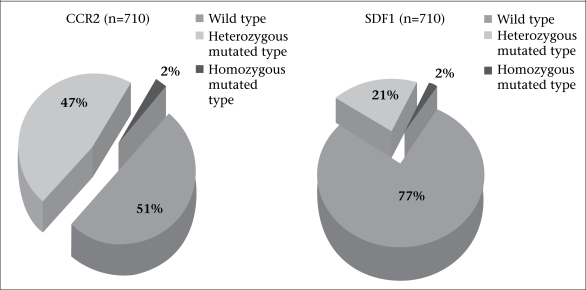
CCR2 and SDF1 genetic mutation status

There was not a single IDU with CCR5 mutant gene. Distribution of the wild type, heterozygous and homozygous mutations in the CCR2 and SDF1 genes in the study population are shown in Fig. 3.

Table 2 shows the seroprevalence of HIV among IDUs with and without the CCR2 and SDF1 mutant genes. However, the difference was not significant (p>0.05).

**Table 2.** Relationship between mutated gene and HIV status

Mutated gene	HIV-positive	HIV-negative	Total no.
CCR2			
Wild type	98 (27)	262	360
Heterozygous mutated type	86 (26)	246	332
Homozygous mutated type	8 (44)	10	18
SDF1			
Wild type	146 (27)	400	546
Heterozygous mutated type	42 (28)	106	148
Homozygous mutated type	4 (25)	12	16

Figures in parentheses indicate percentages

## DISCUSSION

In total, 711 IDUs from four different northeastern states participated in the study. Forty-six percent (n=328) of the participants were aged 20–39 years. The youngest participants (4%; n=29) were aged 19 years or less. HIV seropositivity was increasingly higher with increase in the age of IDUs (Fig. 2). This could be explained by the fact that the older IDUs have a longer duration of injection that could expose them to repeated unsafe injection practices. The unsafe injection practices are associated with transmission of blood-borne infection, including HIV, as evidenced by a number of studies (11, 12).

The results of the study showed that the female IDUs (n=43) had a higher rate of HIV seropositivity compared to the male IDUs (n=668) as consistent with the findings of a study among IDUs in China (13). In the study community, most (90%) female IDUs were sex workers. This fact was also consistent with the findings of several other studies among female IDUs where the seroprevalence of HIV was more among female IDUs compared to males (14–16). This could probably be explained by the dual route of entry of HIV in female IDUs, who were sex workers too. Unsafe sexual activity was a common risk behaviour encountered with them as observed in other studies (17, 18).

Spasmoproxyvon was the highest consumable injectable drug in this part of the country, except parts of Manipur and Nagaland which are traversed by the national highway that acts as the heroin-trafficking route (19, 20). The national highway, which is also considered the heroin-trafficking route, starts from the India-Burma border to Manipur and passes to Nagaland after cutting across the capital city of Manipur, Imphal (21). Consumption of drugs is associated with cultural acceptability, availability, and affordability (11). A study in Bangladesh observed that IDUs usually start addiction with cannabis as the drug of choice and ends up with heroin (22). However, no such diversity in drug-use was observed in the present study.

Unsafe injection-practices are frequently observed in most IDU communities. In this study, HIV seropositivity was observed in 47% (n=177) and 13% (n=44) of the IDUs with always or frequent sharing and occasional or no sharing respectively (Table 1). This difference was significant indicated by OR 5.7. This implies that sharing is associated with higher transmission of HIV. Similarly, sharing of drugs from common ampoules, cleaning water, cotton, etc. were associated with higher transmission of HIV among sharers compared to non-sharers. A similar observation was also made in a study of IDUs in a province in China (23).

Several factors that play an important role in the acquisition of HIV infection include viral load at entry-point (set point), the population density, risk behaviour and cultures of a society, immigration and mixing of population, rate of unemployment and poverty, availability, distribution, and consumption of drugs, and government policies (24). Moreover, about 90% of infected people are not aware that they are carrying the infection, and even if they did, anti-retroviral treatment is not an affordable option for them (18, 25). Apart from these factors, host genetics plays an important role in viral entry giving rise to an epidemic. Results of a study showed that people who are homozygous to CCR5 mutation were protected from HIV-1 infection, and on the other hand, heterozygous state renders partial protection against the infection (26).

The HIV epidemic is spreading rapidly in Europe and North America from Asia and Africa due to mutation of HIV subtypes, intermixing of communities, intravenous drug-use, and commercial sex workers (27, 28). Thus, genetic mutations need to be considered for the analysis of HIV-1 epidemic in a particular region, apart from risk factors and risk behaviours. The mutated gene of CCR5 chemokine co-receptor is a prominent genetic marker of HIV-1 resistance (7). In this study, none of the IDUs had CCR5-mutated gene which is consistent with the finding of another study in diverse populations of Andhra Pradesh, South India (27) and also with the findings of studies in other parts of the world, such as Africa, America, Oceania, South-East Asia, and China (29). As in most parts of the world, the IDUs of northeastern India are not protected by any genetic mutation against HIV infection.

The presence of CCR2 mutation in this study was much higher compared to other studies conducted on diverse populations of Andhra Pradesh, India (29) and in HIV patients of Kuwait (30). On matching unsafe injection-practices, the rate of HIV seropositivity was observed to be almost equal in IDUs with and without CCR2 and SDF1 mutant genes. Since mutation in CCR2 and SDF1 genes slows the progression of HIV, IDUs with absence of these mutated genes might have died earlier than those with mutations. Thus, the former may have been under-represented in this study, leading to a bias in the results. Also, the study includes IDUs who are currently involved in injection-practices. The history of HIV seropositivity of the past IDUs should also be considered to get a true picture of the effect of host genetic mutation on HIV seroconversion and progression. So, the IDUs of northeastern states of India appear to have no additional protection against HIV-1 infection in absence of CCR5 mutation. Regarding the CCR2 and SDF1 genes, further cohort studies are required to understand whether the existing mutations in these genes confer any additional protection against progression of HIV-1.

The present study has explored the seroprevalence of HIV, injecting practices, and risk behaviours of current IDUs in four northeastern states of India where injection-practices are widespread due to the easy availability of drugs. It also explores the existence and magnitude of genetic mutations of chemokine receptors in the IDUs of the northeastern states to determine their genetic protection to HIV infection and progression. It has documented that unsafe injection-practices are widely prevalent in this part of India and shows significant contribution to the spread of HIV infection. The study also revealed that the IDU population of this region is not additionally protected against HIV infection and its progression through genetic mutation of chemokine receptors, such as CCR2-64I, CCR-5 D-32, and SDF-1-3'A. Unlike some parts of the world where such genetic mutation confers certain amount of protection to HIV infection despite high-risk behaviour, the IDUs in the northeastern states of India need to control unsafe injection and sexual practices to decrease the high prevalence of HIV in these states. Also, further in-depth cohort study needs to be conducted to understand the effects of CCR2 and SDF1 mutation on the progression of HIV infection. Such studies can help us further understand the complex relationship among HIV infection, progression of disease, and host genetic diversity.
